# Assessing an isiZulu questionnaire with educators in primary schools in Pietermaritzburg to establish a baseline of knowledge of Autism Spectrum Disorder

**DOI:** 10.1186/s12887-016-0721-8

**Published:** 2016-11-14

**Authors:** Natalie K. Hutton, Carol Mitchell, Mary van der Riet

**Affiliations:** Discipline of Psychology, School of Applied and Human Sciences, University of KwaZulu-Natal, Pietermaritzburg, Private Bag X01, Scottsville, 3209 South Africa

**Keywords:** Autism, Autism Spectrum Disorder, South Africa, Educators

## Abstract

**Background:**

Autism Spectrum Disorder (ASD) is a significant childhood disorder and has a growing prevalence rate across the world. It has been identified in children from a wide range of racial groups, ethnicities and socio-economic groups, making it a globally relevant disorder. However, a lack of research on ASD in Africa makes it difficult to determine the prevalence rate, presentation and level of knowledge regarding the disorder locally. Therefore, assessing knowledge of ASD amongst professionals is a useful starting point for research in countries where research on ASD is limited. Educators in particular are a vital resource due to the likelihood of their early identification of developmental delays in children of school going age. Awareness studies reveal that professionals have poor awareness of ASD and therefore what educators in South Africa know about ASD needs to be established.

**Methods:**

This study translated the Knowledge about Childhood Autism among Health Workers (KCAHW) questionnaire that was originally designed by Bakare and colleagues (Clinical Practice and Epidemiology in Mental Health 4:17, 2008). The isiZulu KCAHW questionnaire was then used to investigate the level of knowledge of ASD amongst educators in Edendale, Pietermaritzburg, South Africa. Fifty (50) educators consented to complete the questionnaire and the data was analysed using the statistical programme SPSS.

**Results:**

The results suggested that educators have an adequate baseline knowledge of ASD but their knowledge was found to be lacking in specific detail. The mean total score for the educator sample was 13.08 (out of a possible 19) which suggested that educators in Edendale, Pietermaritzburg knew 68% of the symptoms covered in the questionnaire.

**Conclusions:**

The *isiZulu* KCAHW questionnaire appears to be a useful measure for use in the South African context. It provided significant information regarding educator knowledge of ASD in Edendale, Pietermaritzburg. However, the analysis also showed that whilst the educators had an adequate general knowledge of ASD, they lacked specific insight into the disorder, particularly with regards to etiology and age of onset. Furthermore, the results showed that there is an opportunity for further research and interventions to develop knowledge of ASD within the local context in South Africa.

**Electronic supplementary material:**

The online version of this article (doi:10.1186/s12887-016-0721-8) contains supplementary material, which is available to authorized users.

## Background

Research shows that Autism Spectrum Disorder (ASD) is the fastest growing childhood disorder in the world [1]. As a result, knowledge about this disorder is progressing as researchers develop a greater understanding of how ASD is viewed, diagnosed and treated across cultures. ASD is a neurologically based developmental disorder that is defined by deficits in social interaction and communication, as well as by the presence of restrictive behaviors [2]. Deficits can be noted across multiple contexts. ASD can usually be identified before the age of three [3] and is almost five times more common in boys than girls [4].

ASD in the DSM 5 is defined by impairments in two specific domains: namely social communication and interaction, and restricted repetitive behaviors [2]. The first major criterion for a diagnosis of ASD is marked deficits in social interaction and communication. This refers to the inability to reciprocate with others on a relational level [2]. Impaired social interaction skills range from a lack of eye contact to an inability to take interest in others [2]. Impaired communication may present in various ways, depending on the child. The second major criterion for a diagnosis of ASD is the presence of restrictive behaviors. This refers specifically to repetitive patterns of behavior, activities and interests [2]. For example, a child may present with the inability to be flexible with routines, or with repetitive hand gestures known as flapping [2, 5]. Children with ASD are commonly fixated on particular interests and develop ritualistic behavioral patterns around said interests [5].

The average prevalence rate of ASD according to the CDC [4] is between 1 and 2% in Asia, Europe and North America, which indicates an increase in ASD cases. In Africa, a preliminary observation done in the 1970s suggested that the rate of ASD was lower than the UK [6]. The data was inconclusive but did provide crucial information that highlighted the presence of ASD in varied geographical regions [6]. No published data on the prevalence rates in Africa exists to date [6]. According to a study conducted by Bakare and Munir [7] only nine articles on ASD in Africa were published between January 2000 and December 2009 [7, 8]. A statistical study on 764 globally published journal articles on ASD confirmed that 78% and 16% were from North America and Europe respectively [9]. Only 4% of the articles represented the rest of the world, which includes Africa [9].

As is the case with Africa as a whole, the prevalence rate of ASD in South Africa is not known [9]. No epidemiological studies have been conducted to date in South Africa and the impact of ASD on the South African population is unknown [10]. If prevalence rates from the United States from 2007, are applied to South Africa, it is possible that 270 000 people in South Africa could have ASD, and this number may be increasing by 5000 cases per year [9]. There was an 8.2% increase in the number of cases presenting with ASD symptoms in Johannesburg between 1996 and 2005 [9].

Although ASD is characterized by particular diagnostic criteria, the presentation of the disorder varies from case to case. Research in the African context has reported the following trends in presentation: A study in Tunisia [11] found that low intellectual functioning was a co-morbid disability in 60% of the African children with autism that they investigated. Bakare and Munir [12] note that there are more non-verbal cases than verbal cases, suggesting that children with autism in Africa are unable to develop language skills [12]. Springer et al. [9] conducted a study to determine how South African children with autism present verbally and found 72% of their cases were non-verbal, confirming previous studies. Given the limited resource context in most sub-Saharan African countries, these trends may be due to the fact that only the more severe cases present for assessment and intervention, whilst children with ASD who are more highly functioning are not diagnosed or assisted and supported.

Despite a growing understanding of ASD, there is wide variation amongst professionals around the world when it comes to knowledge of ASD [1]. An awareness study in Pakistan amongst health workers in Lahore found that there is an incomplete view of autism in Pakistan. The majority of professionals agreed on the basic diagnostic criteria, but not on the age of onset and how autism relates to speech delays. Most of the respondents believed that autism is a temporary childhood disorder. Furthermore, the respondents felt that autism is a form of Schizophrenia [1]. Another study conducted in Pakistan, amongst 170 primary school educators revealed that 55% of the educators knew about ASD as a result of media exposure [13]. Nine percent of educators reported that they had had formal training on ASD [13]. Fifty seven percent of the educators indicated that proper training would be useful for educators [13].

An online survey in the United States of America of parents or caretakers of individuals with autism revealed that pediatricians and health care workers were unable to provide families with the correct information about autism. They discovered that only 20% of the participants found out what they know about autism from professionals; the majority educated themselves about autism via the Internet [14]. A further study in the US involved a survey of educators, and found that their participants’ perceptions of autism were within the average range, whilst their factual knowledge of autism was within the low to average range [15].

A study conducted in Great Britain investigated what educators in both the mainstream (*n* = 102) and special needs (*n* = 40) sectors knew about disorders like ASD [16]. The study found that 99% of the special needs educators and all of the mainstream educators knew what ASD was [16]. Sixty seven percent of special needs educators and 27% of mainstream educators had experience teaching a child with ASD [16]. The most frequently observed features of ASD were social and communication difficulties, obsessive behavior traits, lack of eye contact and the lack of defining physical features [16]. The study concluded that their sample had a reasonably good level of knowledge and awareness of ASD [16].

A study conducted in Singapore amongst 503 educators revealed that 68% of educators had sufficient knowledge of ASD to pass a knowledge assessment test [17]. The study concluded that there were significant deficits in educator knowledge of childhood developmental disorders generally and that the majority of educators possessed a minimum level of knowledge of ASD [17]. Their study also revealed a general lack of knowledge regarding the impact of early intervention for children with ASD [17].

An ASD awareness survey was initiated in Nigeria in 2007 [18]. This revealed that the public had a low level of knowledge of ASD and that health care workers had a low to average level of knowledge and awareness. The results were contrary to global trends of growing ASD awareness [18]. Furthermore, the survey questionnaire used in the study was reportedly not easily understandable or accessible [18]. As a result, Bakare et al. [18] modified the questionnaire and devised the “Knowledge about Childhood Autism among Health Workers (KCAHW) Questionnaire.” The KCAHW questionnaire was devised to achieve two goals: firstly to establish a baseline of knowledge of ASD; secondly to create a tool that could inform education campaigns in Nigeria amongst health care professionals [18].

Increasing public awareness of ASD in South Africa will improve identification of this developmental disorder [9]. Bakare and Munir [7] suggest a school-based awareness study to improve knowledge of ASD. As a result, this research aimed to improve knowledge of ASD by conducting a school-based awareness study focusing on what educators know about ASD.

## Methods

### Location

This study was carried out in two locations in the Pietermaritzburg area in KwaZulu-Natal, South Africa. Firstly, the questionnaire was tested in a higher education context and secondly it was implemented in a ‘township’ context with educators at previously disadvantaged schools. ‘Township’ in the South African context refers to an undeveloped urban area that was previously disadvantaged due to Apartheid policies and is still under resourced. South African schools are categorized according to Quintile ranks, which indicate the degree to which they are resourced by the government. The schools selected for this study were amongst the least resourced schools (quintiles 1, 2 and 3).

### Research design

This research followed a quantitative design and the data collected was numeric and analyzed statistically [19]. The design of this study occurred in three phases:

#### Phase 1

The first step in the research design was to develop a reliable measure of knowledge of ASD. The KCAHW questionnaire devised by Bakare et al. [18] was selected due to recommendations regarding its use in Southern Africa as well as its reliability in the African context. The English KCAHW questionnaire devised by Bakare et al. [18] was translated into *isiZulu*. The questionnaire was translated according to Brislin's [20] and Sperber’s [21] models for translation:

- Translator one translated the English questionnaire into *isiZulu*. Translator two checked the translation and made changes.
- Translator three did a blind back-translation of the *isiZulu* questionnaire back into English.
- Translation A and B were compared. Discrepancies were negotiated with Translator 3 and changes were implemented. Criterion, conceptual and semantic equivalence were assessed. Problem items were revised by the translator and this process was repeated as required until equivalence was gained.
- Following the translation of the questionnaire it was submitted to two experts for scrutiny. One of the experts assessed the isiZulu questionnaire for face validity [22]. The expert psychologist specifically analysed language and cultural discrepancies in the translated questionnaire. The questionnaire was also reviewed by a second expert psychologist for additional consultation, who recommended the inclusion of an introductory question *(“Uyazi yoni ukuthi yini I-Autism?”* Translated: “Do you know what autism spectrum disorder is?”).

#### Phase 2

This involved testing the questionnaire in a pilot test with 50 psychology honors students at UKZN. This pilot study was conducted to assess the reliability of the translated questionnaire prior to using it to assess educators’ knowledge of ASD.

#### Phase 3

The questionnaire was administered to 50 primary school educators in Pietermaritzburg. The data collected from the educator sample was analyzed to assess the extent of knowledge of Autism Spectrum Disorders amongst the educators. This paper specifically reports on the findings of phase three of this study.

### Sampling

This study made use of purposive and convenience sampling. Purposive sampling involves a selected sample group based on theoretical reasons; convenience sampling is based on the accessibility of a sample [19]. Participation was voluntary and there were no perceived risks to the participants.

Fifty participants were sufficient from each site in order to yield enough data to run comparative statistical analyses [23]. Schools were selected based on their participation in a network for under-resourced schools in Pietermaritzburg. These schools were chosen as a result of an already established partnership with the researchers. The schools all came from one geographic area and are classified as Quintile one, two and three schools and are therefore under-resourced. Educators were recruited to join the study by telephone contact with principals of the target schools. A time was arranged for a school visit in order to explain the study. Five schools provided access and staff meetings were arranged on respective days.

### Data collection

Data collection was done using the translated isiZulu KCAHW questionnaire. The questionnaire was a pencil and paper test and took a maximum of 30 min to complete. An explanation of instructions was provided on the questionnaire. Respondents were not asked to supply any identifying details. Participation was voluntary and confidential.

### Data analysis

Educators’ total scores were calculated and individual domain scores were tabulated in the Statistical Package for Social Sciences (SPSS, Version 22). The mean score of the sample population was then calculated in order to determine the average baseline of knowledge across the sample population. The scores for each domain were calculated and analyzed in order to determine whether or not specific domains were more familiar to educators. Educators’ perceptions of ASD with regards to verbal skills were addressed by statistically analyzing the responses to domain two. The data was also analyzed according to descriptive information provided in order to determine whether or not the results are correlated in any way. Age, experience, total scores and answers to the introductory question were correlated with one another.

## Results

A total of 51 educators were recruited from five primary schools in Edendale, Pietermaritzburg (*N* = 51). All questionnaires were completed fully, making them valid for analysis. The youngest respondent was 25 years old and the maximum age was 60 years. The mean age was 46.3 years. All participants were first language *isiZulu* speakers. All participants were black South African. The average number of years of teaching experience amongst the educator sample was 19.29 years. The minimum number of years of experience reported was 1 year and the maximum number of years of experience was 35 years.

### Educator knowledge based on KCAHW scores

**Fig. 1:**
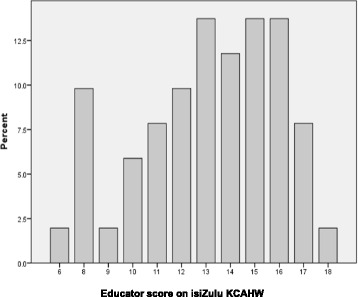
Distribution of educator total scores. This figure shows the distribution of the total scores achieved by the educator sample on the KCAHW questionnaire

Graph 1 shows that 19.6% of educators scored less than ten points on the questionnaire (out of a total of 19). This suggests that 80.4% of educators knew enough features of ASD to score more than 50% on the questionnaire. Just less than half the population scored 13 points or more on the measure (49%). Nineteen educators (23.5%) scored 15 or more and two percent scored above 17 (five educators). This data suggests that most of the educators knew enough about ASD to identify half of the symptoms presented (Fig. 1).

The mean total score for the educator sample was 13.08 out of a possible total of 19. This suggests that the educators scored an average total score of 68% out of a possible 100% and that educators knew approximately 68% of the symptoms covered in the questionnaire. The lowest total score earned was six and the highest was eighteen.

### Pattern of distribution of scores on KCAHW questionnaire

In domain one, which deals with questions on the area of impairments in social interaction, the mean score was 6.47 out of a possible eight which suggests that the educators had a high performance rate on the domain. The majority of educators scored 75% or more on domain one (58.8%). This suggests a high degree of knowledge with regards to ASD symptoms associated with social interaction. More than 60% of the educators answered ‘yes’ for question 1 in domain one (“Marked impairment in use of multiple non-verbal behaviors such as eye to eye contact, facial expression, body postures and gestures during social interaction?”). This suggests that the educators sampled recognize that deficits in eye contact may be associated with ASD, even in the context of different cultural norms around eye contact in South Africa.

In domain two (only one item), the mean score was 0.78 out of a possible score of one which also suggests a high performance rate. Seventy eight percent of the educators scored 100%. This suggests that educators recognize that ASD affects communication and can be associated with a total lack of verbal skills. Less than 20% answered ‘no’ and a small percentage were unsure. With regards to domain two (“Delay or total lack of development of spoken language?”), 40 educators (80%) answered ‘yes’ and therefore stated that ASD is associated with delayed or total lack of speech. Since the question did not separate the two (i.e. a delay or a total lack of skills), it is impossible to make broad conclusions regarding this issue beyond the fact that these educators associate ASD with communication difficulties.

In domain three, the mean score was 3.06 out of a possible four. Twenty five and a half percent of the educators scored at least 50% for the domain. A high proportion of educators scored three or more for the domain (38 educators, 74.5%). Approximately 45% scored four out of four (100%). This suggests that educators recognize that ASD impacts behavior in specific ways.

Domain four assessed knowledge about the etiology of ASD. The mean score for domain four was 2.73 out of a possible six. Five educators scored one or below for this domain. Thirty four educators (66.6%) scored in the below average range of two or three points, and 76.5% scored 50% or less. Eleven educators (21.5%) scored four or above and 5.9% scored above 75%. Refer to Table 1 below for a tabulated version of the data matrices.

**Table 1:** Mean scores for the educator population

Domains	Area of knowledge/symptoms questions addressed	Total score possible	Mean score	Percentage of educators with scores ≥ 75 % correct answers
Domain 1	Impairment in social interaction	8	6.47	58.8 %
Domain 2	Impairment in communication	1	0.78	78.4 %
Domain 3	Obsessive and repetitive behaviours	4	3.06	74.5 %
Domain 4	The nature of ASD and its co-morbidity	6	2.73	5.9 %
Summation of domains 1,2,3 & 4	Summation of scores in the four domains	19	13.08	76.5 %

These results suggest that the educators’ had a high performance rate in domains one, two and three. As stated earlier, 80.4% of educators knew enough features of ASD to score ten or more on the questionnaire. Approximately half the population scored 13 points or more on the measure and approximately 2% scored 17 or higher.

These scores also suggest that domain four was the most challenging for the educators. Domain two had the highest success rate but this is perhaps due to the fact that it was based on only one question. Domain three was the next most successful domain, followed by domain one. This may be in part due to the large number of questions in domain one (eight questions).

### Correlates of knowledge of childhood autism

Correlations highlight the relationship between variables [19]. The educators’ age, years of teaching experience and total scores were analyzed in order to identify any relationship between the variables. Ethnicity and language were not included in this analysis due to the lack of variation across the sample. The correlation analysis did not reveal any significant correlations between the scores and the demographic variables.

## Discussion

The previous studies by Bakare and colleagues [18, 24] documented differing levels of knowledge of autism, depending on their employment context. Those that were based in community mental health contexts had a low level of knowledge of ASD. Their total scores on the KCAHW were 9.6 and 9.8 respectively. This indicates that on average, the community based practitioners knew less than 50% of the symptoms presented in the questionnaire. The healthcare workers based in tertiary healthcare settings had much higher scores (a mean score 12.35). In the current study the educator sample (who are mainly in community based settings) had an average score of 13.08. The data showed that 68% of the educators knew at least 50% of the symptoms of ASD.

It is important to note however that, as in the Singaporean study [17], a sample may still lack general knowledge of ASD despite being able to score 50% or more on the measure. In the current study only 5.9% of the educators scored more than 68% suggesting that whilst the majority of the educators had a general knowledge of ASD, they lacked a specific knowledge of the facts. These studies suggest that professionals may lack detailed knowledge of ASD even if they have general knowledge.

Similar to the study in Pakistan [1], the educators appeared to have an incomplete knowledge of ASD. They could identify the core features but lacked specific knowledge about etiology and co-morbidities in domain four. The lack of knowledge of etiology is not surprising, given that this is still being debated in the field. Domain four was also related to knowledge about the age of onset of ASD. A lack of knowledge in this area may result in missed opportunities for early intervention.

The studies mentioned earlier generally revealed that on average, professionals have low levels of knowledge of ASD. This study showed that 80.4% of educators knew enough features of ASD to score more than 50% on the questionnaire. This may be, in part, due to the nature of their profession. Educators are exposed to childhood disorders and are perhaps expected to have a working knowledge of the disorders. Furthermore, the educators were sampled from schools that belong to a support network. This means that at least one educator in each school has regular training on education-related topics. This educator often disseminates that information to the staff at his/her school, which suggests that the educators have secondary access to training on childhood disorders on a frequent basis. York et al. [16] sampled special needs educators as well as mainstream educators and found that their knowledge was reasonable. The sample of educators in this study performed similarly and was also comprised of mainstream educators as well as those with access to informal special needs training.

The results suggest that the educator population has baseline knowledge of ASD. The educators appeared to perform with an even distribution across variables such as age and work experience. These did not seem to impact on their knowledge of ASD in any way. The inclusion of questions such as, “Have you had any training in ASD?” or “Have you ever worked with a child with ASD?” may prove useful in future studies, as this was found to correlate with knowledge of autism in other studies [24].

### Knowledge of specific items

The issue of verbal skills in children with ASD was raised above, with particular reference to findings in the African context. The literature states that African children typically present with non-verbal ASD [7]. However, this may be due to more identifiable atypical development in non-verbal children that results in a higher referral and reporting rate. Whilst this critique cannot be validated, it raises an interesting question with regards to how educators might perceive ASD in Africa. Eighty percent of the educators in this study stated that ASD is associated with delayed or total lack of speech. This suggests that the educators recognise that ASD is associated with a speech deficit of variable degree, not only a total lack of speech. The complication with this question is that it included both the notion of a delay or a total lack of skills which makes it difficult to draw conclusions regarding this issue. Future research using this scale could be improved by including more than one item in this domain and avoiding double questions such as this item.

ASD detection may be complicated by African cultural practices of not making eye contact with an elder. It is possible that as Zulu children do not make eye contact with their elders perhaps this symptom of ASD is not recognized within this context [25]. Question one (“Marked impairment in use of multiple non-verbal behaviors such as eye to eye contact, facial expression, body postures and gestures during social interaction?”) assessed knowledge of this symptom. The data suggests that more than 60% of the educators recognize that non-verbal behaviors are impaired in ASD. Given that the questionnaire item included many non-verbal behaviors, what the educators consider symptomatic regarding eye contact specifically cannot be determined further at this stage.

### Limitations of the current study

The original study by Bakare et al. [18] produced a high Cronbach’s alpha score (.97). The current study generated a much lower Cronbach’s alpha value and as a result, the reliability of the translated measure appears to be lower than the original questionnaire (Cronbach's alpha for the educator population was .60). This is compounded by the fact that the language of choice for this study was *isiZulu,* a complex language due to its predominantly oral history. This is particularly true for written and technical medical terms such as “auto-immune” which are either unknown or discussed by way of code-switching in everyday language.

Secondly, the sample size was small [1]. Whilst it allowed for valuable data to be produced, a larger sample size may have provided more insight into the reliability of the translated measure and possibly added to the generalizability of the results. Furthermore, the educators were recruited through an educator support network, which may have skewed the results to a degree.

Additionally, the wording of the questions could have impacted on the results as this may have caused the educators to answer in the affirmative [1]. Some questions may have constructed the behaviors as negative and this may have impacted on validity. For example, domain 1 question 8 states, “Social smile is usually absent in a child with autism.” Instead, the question could be worded as follows, “A social smile is present in a child with autism.” Furthermore, the questions could list symptoms related to a social smile. This could increase the variability in the responses and thereby reduce the possibility of set response styles.

Finally, relying solely on the KCAHW questionnaire to assess educators’ knowledge of ASD may have limited the results. Another instrument could have yielded different results or may have provided additional information to further develop the current understanding of knowledge of ASD amongst educators. However, no other instruments have been recommended for use in sub-Saharan Africa and as a result, this instrument was the most applicable to the context.

### Future research

Future research could focus on other translations of the instrument. It is worth noting that the participants in this study indicated that they would have preferred a bilingual questionnaire – in both English and *isiZulu.* Various adaptations to the structure of the questionnaire could also be investigated (e.g. requesting demographic details at the end, using Likert scale answer formats; including more items in Domain two; providing space for qualitative comments on the form; collapsing the domains etc.). The questionnaire could be updated to reflect the new criteria (DSM 5) and include various symptoms across the spectrum. For example, not all children with ASD have delayed speech. In addition the questionnaire should be updated to reflect the new terminology in the DSM 5, e.g. Domain 4, “mental retardation” is now “intellectual disability”. Also, in Domain 4, changing item vi regarding the onset of autism to include some age spans may provide more detailed information on early identification. DSM 5 criteria for ASD are categorized differently from the DSM-IV-TR that the KCAHW questionnaire is modelled on. Thus collapsing the domains would be more consistent with the DSM 5. Lastly, the inclusion of criteria that are *atypical* of ASD in the questionnaire may better determine respondents’ knowledge of ASD.

## Conclusion

In conclusion, the *isiZulu* KCAHW questionnaire appears to be a useful measure for use in the South African context. It provided significant information regarding educator knowledge of ASD in Edendale, Pietermaritzburg, revealing that these educators lack knowledge regarding the etiology of ASD and age of onset. This research identified that educators would benefit from training on ASD in order to assist them with early identification of the disorder and how to proceed thereafter. Further research is also recommended with translations and adaptations of the instrument for other contexts in sub-Saharan Africa.

## Additional files

Additional file 1: Knowledge about Childhood Autism among Health Workers (KCAHW) Questionnaire. (DOCX 12 kb)
Additional file 2: isiZulu KCAHW questionnaire. (DOCX 18 kb)

## Abbreviations

ASD: Autism Spectrum Disorder
DSM 5: Diagnostic and Statistical Manual of Mental Disorders, 5th edition
DSM-IV-TR: Diagnostic and Statistical Manual of Mental Disorders, 4^th^ edition revised
KCAHW: Knowledge of childhood autism amongst health workers
