# Supplementary material for: Assessing an isiZulu questionnaire with educators in primary schools in Pietermaritzburg to establish a baseline of knowledge of Autism Spectrum Disorder
Source: BMC Pediatr. 2016 Nov 14;16:185. doi: 10.1186/s12887-016-0721-8 (PMC5109657; doi:10.1186/s12887-016-0721-8)
Supplement: Additional file 1: — Knowledge about Childhood Autism among Health Workers (KCAHW) Questionnaire. (DOCX 12 kb) [file 12887_2016_721_MOESM1_ESM.docx]

**Appendix 1: Original KCAHW questionnaire by Bakare et al. [18]**

Please do not consult formal text books to answer these questions.

Thank you for your time.

The following behaviours best describe a child with Childhood Autism:

Domain 1

i. Marked impairment in use of multiple non-verbal behaviours such as eye to eye contact, facial expression, body postures and gestures during social interaction?

(A) Don't Know, (B) Yes, (C) No

ii. Failure to develop peer relationship appropriate for developmental age?

(A) Don't Know, (B) Yes, (C) No

iii. Lack of spontaneous will to share enjoyment, interest or activities with other people?

(A) Don't Know, (B) Yes, (C) No

iv. Lack of social or emotional reciprocity?

(A) Don't Know, (B) Yes, (C) No

v. Staring into open space and not focusing on any thing specific?

(A) Don't Know, (B) Yes, (C) No

vi. The child can appear as if deaf or dumb?

(A) Don't Know, (B) Yes, (C) No

vii. Loss of interest in the environment and surroundings?

(A) Don't Know, (B) Yes, (C) No

viii. Social smile is usually absent in a child with Autism?

(A) Don't Know, (B) Yes (C) No

Domain 2

i. Delay or total lack of development of spoken language?

(A) Don't Know (B) Yes (C) No

Domain 3

i. Stereotyped and repetitive movement (e.g. Hand or finger flapping or twisting)?

(A) Don't Know (B) Yes, (C) No

ii. May be associated with abnormal eating habit?

(A) Don't Know, (B) Yes, (C) No

iii. Persistent preoccupation with parts of objects?

(A) Don't Know, (B) Yes, (C) No

iv. Love for regimented routine activities?

(A) Don't Know, (B) Yes, (C) No

Domain 4

i. Autism is Childhood Schizophrenia?

(A) Don't Know, (B) Yes, (C) No

ii. Autism is an auto-immune condition?

(A) Don't Know, (B) Yes, (C) No

iii. Autism is a neuro-developmental disorder?

(A) Don't Know, (B) Yes, (C) No

iv. Autism could be associated with Mental Retardation?

(A) Don't Know, (B) Yes, (C) No

v. Autism could be associated with Epilepsy?

(A) Don't Know, (B) Yes, (C) No

vi. Onset of Autism is usually in,

1. Neonatal age, (B) Infancy, (C) Childhood
