# Supplementary material for: Assessing an isiZulu questionnaire with educators in primary schools in Pietermaritzburg to establish a baseline of knowledge of Autism Spectrum Disorder
Source: BMC Pediatr. 2016 Nov 14;16:185. doi: 10.1186/s12887-016-0721-8 (PMC5109657; doi:10.1186/s12887-016-0721-8)
Supplement: Additional file 2: — isiZulu KCAHW questionnaire. (DOCX 18 kb) [file 12887_2016_721_MOESM2_ESM.docx]

**Appendix 2: isiZulu KCAHW questionnaire**

***isiZulu* Knowledge about childhood Autism amongst health workers questionnaire**

**Ngiyacela ugcwalise okulandelayo ukuze usize kulolucwaningo:**

- Ngineminyaka ewu __________yobudala
- Sengifundise iminyaka ewu_________________
- Ngi- ☐ Mnyama ☐ Indiya ☐ Ikhaladi ☐ Mhlophe
- Ngiyakhuluma ☐ *isiZulu* ☐ *isiXhosa* ☐ *isiNgisi* ☐ *isiBhunu*

**Imiyalelo:**

Imibuzo ngoLwazi lwaBasebenza ngeZempilo mayelana ne “Autism” kubantwana

**Zungeleza impendulo efaneleyo**

- **Isibonela**

**Inyanga yokuqala onyakeni uMasingana**

☐ Angazi  Yebo ☐ Cha

Ngiyabonga ngesikhathi sakho.

__________________________________________________________

**Uyazi yoni ukuthi yini I-Autism?**

**☐ Yebo ☐ Cha**

**Isiqephu 1**

**Zungeleza impendulo efaneleyo. Lezi zindlela zokuziphatha zichaza ingane ene “Autism”:**

1. Ukungakwazi ukusebenzisa izindlela eziningi ngaphandle kokukhuluma njengokubhekana emehlweni, umumo wobuso (*facial expression*), ukumiswa komzimba nokunqathuzisa izandla uma kuthintanwa.

☐ Angazi ☐ Yebo ☐ Cha

1. Ukuhluleka nokuba nobudlelwane bontanga obufanele lobo budala?

☐ Angazi ☐ Yebo ☐ Cha

1. Ukukhombisa ukuthi akafuni ukujabula nabanye, ukuba nomnako noma ukwenza izinto nabanye?

☐ Angazi ☐ Yebo ☐ Cha

1. Ukuswela isenaniselo semizwa nangokuzwelana nabanye?

☐ Angazi ☐ Yebo ☐ Cha

1. Ukugqolozela obaleni engabukisisi lutho?

☐ Angazi ☐ Yebo ☐ Cha

1. Kungathi ingane iyisithulu noma isimungulu?

☐ Angazi ☐ Yebo ☐ Cha

1. Ukungabi nogqozu ngenhlalo yendawo yakhe?

☐ Angazi ☐ Yebo ☐ Cha

1. Kujwayelekile ukuthi akukho ukumamatheka enganeni uma kukhona abanye? (Isibonela: ingane iyamamatheka uma kumamatheka abanye noma iculelwa?)

☐ Angazi ☐ Yebo ☐ Cha

**Isiqephu 2**

**Zungeleza impendulo efaneleyo. Lezi zindlela zokuziphatha zichaza ingane ene “Autism”:**

1. Ukuphuza kokuqhubeka ukukhuluma noma kungekho ukuqhubeka kokukhuluma?

☐ Angazi ☐ Yebo ☐ Cha

**Isiqephu 3**

**Zungeleza impendulo efaneleyo. Lezi zindlela zokuziphatha zichaza ingane ene “Autism”:**

1. Izindlela zokunyakaza ziyaphindwa noma zihlala zifana ( isib. Ukugwincigwinciza isandla noma iminwe)

☐ Angazi ☐ Yebo ☐ Cha

1. Kungahlanganiswa nezindlela zokudla ezingajwayelekile

☐ Angazi ☐ Yebo ☐ Cha

1. Uhlala elibaziseke ngezingxenye zezinto. Isibonelo: Unaka amasondo emoto hhayi imoto yonke.

☐ Angazi ☐ Yebo ☐ Cha

1. Ukuthanda impikelelwana yezenzo, zihlala zenziwa ngendlela efanayo, engejabuli umazishintja?

☐ Angazi ☐ Yebo ☐ Cha

**Isiqephu 4**

**Zungeleza impendulo efaneleyo**

**Lezi zindlela zokuziphatha zichaza ingane ene “Autism”:**

1. I-Autism ingukuba umntwana onezimilozimbili (Schizophrenia)?

☐ Angazi ☐ Yebo ☐ Cha

1. I-Autism kusho ukuba nesifo lapho umzimba uzilwelayo esibizwa nge-auto-immune?

☐ Angazi ☐ Yebo ☐ Cha

1. I-Autism yisimo senkinga nokukhula kwemizwa?

☐ Angazi ☐ Yebo ☐ Cha

1. I-Autism ingaba ihlangene nokukhubazeka kwengqondo

☐ Angazi ☐ Yebo ☐ Cha

1. I-Autism ingaba ihlangene nesithuthwane (isifo sokuwa)

☐ Angazi ☐ Yebo ☐ Cha

1. Ukuqala kwe-Autism kujwayele ukuba esikhathini

☐esingaphambi kokuzalwa ☐sobuntwana (izinyanga months 0-24)

☐sobungane (iminyaka years 2-18)

_____________________________________________________________________

**Ngiyabonga Kakhulu.**
